# Novel *Mycobacterium tuberculosis* Complex Pathogen, *M. mungi*

**DOI:** 10.3201/eid1608.100314

**Published:** 2010-08

**Authors:** Kathleen A. Alexander, Pete N. Laver, Anita L. Michel, Mark Williams, Paul D. van Helden, Robin M. Warren, Nicolaas C. Gey van Pittius

**Affiliations:** Virginia Polytechnic Institute and State University, Blacksburg, Virginia, USA (K.A. Alexander, P.N. Laver); Centre for Conservation of African Resources; Animals, Communities and Land Use, Kasane, Botswana (K.A. Alexander, P.N. Laver); ARC-Onderstepoort Veterinary Institute, Pretoria, South Africa (A.L. Michel); University of Pretoria, Pretoria (A.L. Michel, M. Williams); Stellenbosch University, Tygerberg, South Africa (P.D. van Helden, R.M. Warren, N.C. Gey van Pittius)

**Keywords:** Tuberculosis, Mycobacterium tuberculosis complex, Mycobacterium mungi, banded mongoose, human–wildlife interface, wildlife, dassie bacillius, tuberculosis and other mycobacteria, dispatch

## Abstract

Seven outbreaks involving increasing numbers of banded mongoose troops and high death rates have been documented. We identified a *Mycobacterium tuberculosis* complex pathogen, *M. mungi* sp. nov., as the causative agent among banded mongooses that live near humans in Chobe District, Botswana. Host spectrum and transmission dynamics remain unknown.

A previously unidentified *Mycobacterium tuberculosis* complex pathogen has emerged in banded mongooses (*Mungos mungo*) in Botswana; we named the pathogen mongoose bacillus, or *M. mungi* sp. nov. This pathogen causes high mortality rates among banded mongooses that live in close association with humans because these animals live in human-made structures and scavenge human waste, including feces.

Banded mongooses are social, fossorial, viverids that feed on invertebrates and small mammals including subterranean species (*1*). We initially identified tuberculosis (TB) disease in banded mongooses in 2000. The outbreak appeared to spread as a point-source infection between mongoose troops living in close association with humans and human waste; infection spread through towns and the associated national park (*2*). During 2000–2010, a total of 7 outbreaks occurred (increasing in duration), mongoose troop involvement increased, and the spatial and temporal connection between cases decreased. Infected mongoose troops are now widely identified across the landscape, including protected areas and urban centers (Figure 1), and high mortality rates threaten the survival of smaller troops. In this study area of Chobe District, Botswana, TB has been identified in only humans and mongooses. Strain assessment of human TB has not been conducted; the full host spectrum and transmission dynamics of this pathogen, currently unknown, are the focus of our ongoing research.

**Figure 1:**
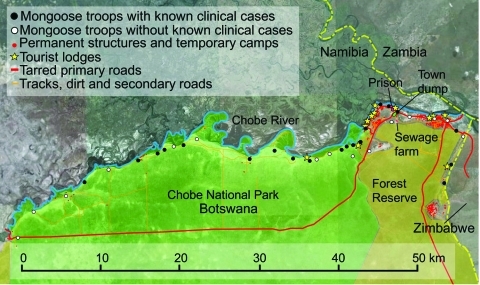
Locations of infected and unaffected banded mongoose troops and human infrastructural development, Chobe District, Botswana.

## The Study

During 2000–2010 in Chobe District, Botswana, we performed 38 necropsies on macroscopically TB-positive mongooses, of which 18 were further evaluated and TB was confirmed by histopathologic examination. An in-depth histologic evaluation was performed on a subsample of 8 of these animals from the 2008 outbreak. The most striking feature identified in the sick mongooses was anorexia, followed by nasal distortion and, less commonly, erosions of the nasal planum with involvement of the hard palate. For 7 of the 8 TB-positive animals examined intensively, macroscopic lesions were noted on the nasal planum. Histologic examination detected unequivocal TB lesions in the skin of the nose and the anterior nasal mucosa. Our findings suggested entry of the organism through erosions on the nasal planum, perhaps in association with abrasions, which might occur during foraging. Such lesions were present in the hairless parts of the nose tip of most TB-infected mongooses. Furthermore, granulomatous inflammation and mycobacterial organisms were found in the dermis of the skin directly below these erosions. Inflammation and organisms were present in some cases in the nasal mucosa, but erosion was not found. Thus, organisms could not have been in the lumen of the nasal cavity. This finding is consistent with pneumonic TB being present in only a few advanced cases of disseminated TB. This pattern was consistent among all animals examined postmortem during the study period. Histologically, the TB pneumonia was determined to be hematogenous rather than bronchogenous (i.e., by inhalation); thus, no evidence for aerosol transmission was found. Rather, pathogen invasion appears to have occurred through the nasal planum of the mongoose, and hematogenous or lymphatic spread through the body was a strikingly unique feature of this particular *M. tuberculosis* complex organism.

Samples from histologically positive mongooses were positive by PCR for the MPB70 target, IS*6110* element, and 16S rDNA, indicating that the infective organism was a member of the *M. tuberculosis* complex (*3**,**4*). Samples were further evaluated with an *M. tuberculosis* complex–specific multiplex PCR (*5*), which provided distinct results, differing clearly from those for other members of the *M. tuberculosis* complex (Table 1).

**Table 1:** Genomic regions of difference of *Mycobacterium tuberculosis* complex members compared with M. mungi*

*Mycobacterium* species	Region of difference					
	RD1^BCG^	RD4	RD9	RD12	RD1^mic^	RD2^seal^
*M. canettii*	P	P	P	A	P	P
*M. tuberculosis*	P	P	P	P	P	P
*M. africanum*	P	P	A	P	P	A
*M. microti*	P	P	A	P	A	P
*M. pinnipedii*	P	P	A	P	P	A
*M. caprae*	P	P	A	A	P	P
*M. bovis*	P	A	A	A	P	P
*M. bovis* BCG	A	A	A	A	P	P
*M. mungi*	P	P	A	P	P	P

*Determined by PCR amplification (*5*). RD, region of difference; RD1^BCG^, *M. bovis* BCG–specific RD1; RD1^mic^, *M. microti*–specific RD1; RD2^seal^, *M. pinnipedii*–specific RD2; P, present; A, absent.

**Figure 2:**
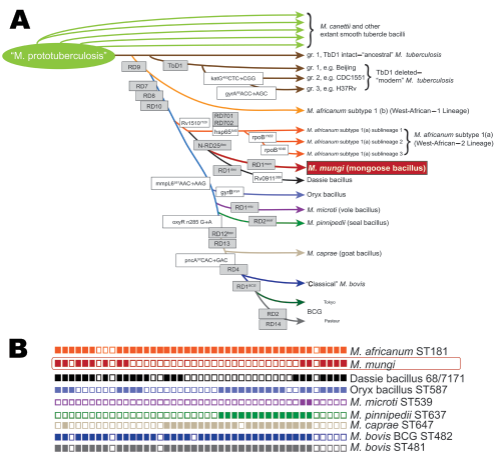
A) Schematic of the phylogenetic relationships among *Mycobacterium tuberculosis* complex species, including newly discovered *M. mungi,* based on the presence or absence of regions of difference (gray boxes) as well as specific single-nucleotide polymorphisms (white boxes), modified from (*7*). B) Spoligotype of *M. mungi* compared with representative spoligotypes from other *M. tuberculosis* complex species (*8*–*10*).

The *gyrB* gene (encoding for gyrase B) sequence, used to identify *M. tuberculosis* complex member–specific sequence single-nucleotide polymorphisms (SNPs) (*6*), identified the position of the organism as being situated between dassie bacillus and *M. africanum* subtype 1(a) and showed no detectable new SNPs (Figure 2, panel A). Amplification of RD701 and RD702 and lack of SNPs in *rpoB* and *hsp65* genes demonstrated that this organism was not a member of the *M. africanum* subtype 1(a) sublineage (*6**,**11*) (Figure 2, panel A).

Three markers were evaluated to definitively exclude the organism from being dassie bacillus: N-RD25^das^ deletion, RD1^das^ deletion, and SNP 389 in the gene Rv0911 (*6*). The N-RD25^das^ amplification gave the right product for the presence of a deletion in this region, and further sequencing confirmed that it contained a deletion in the same position as N-RD25^das^; however, sequencing of Rv0911 showed no SNP at position 389, indicating that this organism was not dassie bacillus. As a final test, we amplified the RD1^das^ region but were unable to amplify a product from the mongoose isolates. We redesigned primers to amplify a smaller region of different diagnostic sizes (248 bp when RD1^das^ is deleted and 318 bp when RD1 is intact) but still had no amplification. This finding indicates that the RD1 region is deleted in this organism but that the deletion is larger than that of the dassie bacillus.

We then used spoligotyping analysis (*12*) to further evaluate mongoose samples and identified a unique spoligotype pattern with no known matches in the international spoligotyping database SpolDB4 (*9*) or the *M. bovis*–specific spoligotype database (www.Mbovis.org) (Figure 2, panel B). This pattern was constant during 2000–2009 in different mongoose troops and locations (Table A1). This unique spoligotyping pattern will enable identification of *M. mungi* in future TB surveillance programs.

For these same isolates, the full set of 24 mycobacterial interspersed repetitive unit–variable number tandem repeats (*13*) identified a pattern that was unique compared with others in the international database at www.miru-vntrplus.org (Table 2). Our examination also included dassie bacillus, which had not been previously analyzed. Evidence of multiple *M. mungi* substrains circulating between years and within social groups (6601B and 6600B) in the same outbreak year (Table 2) suggests complexity in *M. mungi* transmission and potential evolution of the organism over the past decade.

**Table 2:** Comparison of full 24-set MIRU-VNTR of selected *Mycobacterium mungi* isolates*

Sample no.	1883	6601B	6606A	6600B	6875	–	8163/02	24	287/99	7739/01	5358/99	8490/00	H37Rv
Year isolated	2000	2002	2002	2002	2008	–	–	–	–	–	–	–	–
Species	*M. mungi*	*M. mungi*	*M. mungi*	*M. mungi*	*M. mungi*	DB	*M. a.*	OB	*M. microti*	*M. p.*	*M. caprae*	*M.* *bovis*	*M. t.*
MIRU 02	2	2	2	2	2	2	2	2	2	2	2	2	2
VNTR 0424/ Mtub04	3	3	3	3	3	2	4	2	3	3	4	2	2
VNTR 0577/ ETR-C	3	3	3	3	3	5	5	5	5	4	5	5	4
MIRU 04/ ETR-D	3	3	3	3	3	3	2	3	4	5	3	4	2
MIRU 40	1	1	1	1	1	2	2	2	2	2	2	2	1
MIRU 10	5	5	5	5	5	7	7	7	5	6	6	2	3
MIRU 16	3	3	3	3	3	3	4	4	6	4	2	4	2
VNTR 1955/ Mtub21	3	3	3	3	3	3	4	3	3	4	3	3	2
MIRU 20	2	2	2	2	2	2	2	2	1	2	2	2	2
VNTR 2163b/ QUB11b	0	No	No	No	No	7	5	No	6	9	4	4	5
VNTR 2165/ ETR-A	6	6	No	6	6	6	6	3	9	9	5	4	3
VNTR 2347/ Mtub29	3	3	3	3	3	3	3	3	3	3	3	3	4
VNTR 2401/ Mtub30	4	4	4	4	4	3	4	4	4	4	4	4	2
VNTR 2461/ ETR-B	4	4	4	4	4	4	4	2	3	3	3	3	3
MIRU 23	4	4	4	4	4	4	4	4	4	4	4	4	6
MIRU 24	2	2	2	2	3	2	2	1	2	2	2	1	1
MIRU 26	4	4	3	3	4	5	4	4	2	2	4	3	3
MIRU 27	3	3	3	3	3	4	3	3	2	2	3	3	3
VNTR 3171/ Mtub34	3	3	3	3	3	3	3	3	3	3	2	3	3
MIRU 31/ ETR-E	8/9	8/9	8/9	8/9	8/9	5	5	4	1	3	5	3	3
VNTR 3690/ Mtub39	2	No	No	No	No	5	4	4	3	3	1	2	5
VNTR 4052/ QUB26	No	No	No	No	No	4	6	2	9	7	3	5	5
VNTR 4156/ QUB4156	No	No	No	No	No	3	3	3	3	0	3	1	2
MIRU 39	2	2	2	2	2	2	2	2	2	2	2	2	2

*MIRU, mycobacterial interspersed repetitive unit; VNTR, variable number tandem repeats; DB, Dassie bacillus; *M. a., M. africanum*; OB, Oryx bacillus; *M. p.,* *M. pinnipedii*; *M. t.,* *M. tuberculosis*; –, not applicable.; no, no amplification. Gray shading highlights differences between the *Mycobacterium tuberculosis* complex species and *M. mungi* as well as variation within *M. mungi* samples evaluated. DB data supplied by S. Parsons. OB data supplied by P. Supply. *M. africanum* West African 2 strain 8163/02 (ST181), *M. microti* strain 287/99 (ST539), *M. pinnipedii* strain 7739/01, *M. caprae* strain 5358/99 (ST647), and *M. bovis* strain 8490/00 (ST482) data from www.miru-venterplus.org.

## Conclusions

This newly identified mycobacterial pathogen has many unique ecologic characteristics that set it apart from other members of the *M. tuberculosis* complex. First, it causes high numbers of deaths of banded mongooses, threatening local extinction of smaller social groups. Second, rather than having a primary respiratory transmission route with direct transmission between individuals, as is characteristic of other *M. tuberculosis* complex species, *M. mungi* appears to infect banded mongooses by means of a nonrespiratory route through the nasal planum, suggestive of environmental transmission. Third, the time from clinical presentation to death for affected mongooses is generally short (2–3 months) compared with that for other *M. tuberculosis* complex pathogens (more chronic infection, can take years to progress to death). Acute illness and high mortality rates, as seen in banded mongooses with *M. mungi* infection, have been associated with extremely isolated human communities newly exposed to TB (*14*).

Conventional laboratory culture, biochemical testing, and a limited molecular evaluation were insufficient for differentiating *M. mungi* from *M. tuberculosis (**2**).* Organism differentiation required an extensive suite of additional molecular assessments not available at that time, thus underscoring the difficulty of diagnosing *M. tuberculosis* complex agents correctly and the inability of most national health laboratories to do so. The fact that new host-adapted *M. tuberculosis* complex species continue to be identified illustrates the diversity within the *M. tuberculosis* complex and stresses the need for sensitive techniques for species differentiation. The identification of this previously unknown pathogen within the *M. tuberculosis* complex identifies new concerns for human and animal health and illustrates the continuing scope of the threat posed by TB pathogens.

## Appendix

**Table A1:** Spoligotyping results for selected samples from 2000 to 2009

Sample no	Animal ID	Year	Month	Troop	Spoligotype
1882B	BM2344	2000	August	CSL	Mongoose bacillus type
1883	BM2345	2000	August	MSL	Mongoose bacillus type
6601B	BM M2F	2002	May	CGL	Mongoose bacillus type
6606A	BM M5M	2002	May	CGL	Mongoose bacillus type
6599D	BM203	2003	June	CL	Mongoose bacillus type
6600B	BM204	2004	September	CL	Mongoose bacillus type
6875	BM2308	2008	May	MSL	Mongoose bacillus type
8809	BM8809	2009	June	SEF	Mongoose bacillus type
