# Correction Vol. 16, No. 8

**DOI:** 10.3201/eid1612.C11612

**Published:** 2010-12

**Keywords:** errata. Erratum, Correction Vol. 16, No. 8

Table 2 in the article Novel *Mycobacterium tuberculosis* Complex Pathogen, *M. mungi* (K.A. Alexander et al.) contained several errors related to scoring of mycobacterial interspersed repetitive unit–variable number tandem repeats of selected isolates. The article has been corrected online (http://wwwnc.cdc.gov/eid/article/16/8/10-0314_article.htm). 

